# Chronic alcohol withdrawal-associated increases in VTA *Hcrtr1* expression are associated with heightened nociception and anxiety-like behavior in female rats

**DOI:** 10.3389/adar.2025.14199

**Published:** 2025-04-03

**Authors:** Shealan Cruise, Maria E. Secci, Leslie K. Kelley, Nathan M. Sharfman, Keishla Rodriguez-Graciani, Tiffany A. Wills, Nicholas W. Gilpin, Elizabeth M. Avegno

**Affiliations:** ^1^ Department of Physiology, Louisiana State University Health Sciences Center, New Orleans, LA, United States; ^2^ Department of Cell Biology and Anatomy, Louisiana State University Health Sciences Center, New Orleans, LA, United States; ^3^ Alcohol and Drug Center of Excellence, School of Medicine, Louisiana State University Health Sciences Center, New Orleans, LA, United States; ^4^ Neuroscience Center of Excellence, School of Medicine, Louisiana State University Health Sciences Center, New Orleans, LA, United States; ^5^ Southeast Louisiana VA Healthcare System (SLVHCS), New Orleans, LA, United States

**Keywords:** alcohol, nociception, female, orexin, ventral tegmental area, anxiety-like behavior, withdrawal

## Abstract

Alcohol withdrawal is characterized by various symptoms that include pain and negative affect in the absence of the drug. The neural underpinnings of these behaviors are not entirely understood, but orexin has emerged as a candidate target for the treatment of substance use disorders. Here, we explored changes in orexin system-related gene expression in brain regions important for mediating reward and stress, including the ventral tegmental area (VTA) and extended amygdala (including the central amygdala, nucleus accumbens shell, and bed nucleus of the stria terminalis), in adolescent and adult female Wistar rats following chronic alcohol exposure. We observed higher numbers of *Hcrtr1*- (orexin receptor 1)-expressing neurons in the VTA of adolescent and adult female rats during withdrawal from chronic alcohol exposure. The number of *Hcrt1*+ VTA neurons was negatively correlated with thermal sensitivity in adolescent female rats and anxiety-like behavior in adult female rats. These data suggest that chronic alcohol effects on orexin receptor expression in the VTA are related to specific behaviors that manifest during withdrawal, highlighting potential avenues for targeting alcohol withdrawal-associated behaviors across development.

## Introduction

Alcohol use disorder (AUD) is a prevalent condition, affecting over 29 million Americans [1]. Among the clinical diagnostic criteria for AUD are physiological adaptations to alcohol use (e.g., presence of tolerance or withdrawal symptoms [2]), and these can drive continued alcohol use in dependent individuals in an effort to reduce symptomatology. Alcohol misuse and AUD prevalence has increased among women in recent years, reducing the size of the gap between women and men in these statistics, and stimulating interest in sex differences in AUD biology and behavior [3–5]. Of particular concern is adolescent drinking, which is a significant risk factor for later-life AUD development [6]. Recent work points to an increase in initiation of drinking [7], faster progression to heavy drinking episodes [8], and higher rate of alcohol misuse-related emergency department visits [9] among females during early adolescence compared to males. Collectively, these trends highlight the importance of including females in preclinical studies. Here, we investigated the effects of chronic alcohol exposure during two time points (adolescence and adulthood) on behavioral outcomes and region-specific gene expression in female Wistar rats.

Withdrawal symptoms, including negative affect and hyperalgesia, can facilitate relapse in individuals with AUD. While the neurobiology underlying alcohol withdrawal-related behaviors is not entirely understood, brain regions thought to be key for mediating those behaviors include the ventral tegmental area (VTA) in the midbrain, as well as the central amygdala (CeA), nucleus accumbens shell (NAc_shell_), and bed nucleus of the stria terminalis (BNST) [10] in the forebrain. Interestingly, there are robust reciprocal connections between the VTA and extended amygdala subregions, neurons in both regions are activated by acute and chronic alcohol exposure, and VTA-extended amygdala circuits display increased activity following acute/sub-chronic (e.g., VTA→NAc, BNST↔VTA, and CeA→VTA) and chronic (e.g., VTA→CeA) alcohol exposure [11].

Subregions of the extended amygdala are critical for mediating anxiety-like behavior and hyperalgesia during alcohol withdrawal in rodents [12]. For example, chronic alcohol exposure leads to anxiety-like behavior during withdrawal, an effect attenuated by pharmacological manipulation of NAc_shell_ signaling in adolescent or adult male mice [13, 14], BNST signaling in adult male rats [15] and mice [16], and CeA signaling in adult male rats [17, 18]. Similarly, chronic alcohol exposure produces thermal and mechanical hyperalgesia during withdrawal, which is reversed by pharmacological and optogenetic manipulation of CeA neurons in adult male rats [19], as well as pharmacological alteration of BNST [20] and NAc_shell_ [21] signaling in male and female mice and rats following adolescent alcohol exposure. Here, we analyzed expression patterns of genes within each region after chronic alcohol exposure during adolescence and adulthood.

We have previously demonstrated increased activity in a subset of VTA neurons of adult male rats during withdrawal from chronic alcohol exposure, as measured by Fos expression and spontaneous neural activity [22]. The mechanism by which this cellular activation occurs is unknown, but one possibility is via signaling of orexin (also known as hypocretin), a neuropeptide that is released into the VTA from lateral hypothalamus (LH) inputs, has a key role in numerous homeostatic and reward functions [23], and has an excitatory effect on VTA DA neurons when bath applied *in vitro* [24]. Orexin can therefore modulate the activity of projections (e.g., dopaminergic) from the VTA to regions in the extended amygdala; additionally, regions within the extended amygdala express orexin receptors and receive innervation directly from the hypothalamus [25]. Orexin signals through specific receptors, including the orexin-1 receptor (OX1R) and orexin-2 receptor (OX2R). OX1R activation has an excitatory effect on VTA neurons via CB1R-mediated disinhibition (i.e., 2-AG synthesis occurs downstream of OX1R activation, which then activates CB1Rs on presynaptic GABAergic neurons, thereby reducing inhibitory tone onto VTA projection neurons) [24]. In humans, blood orexin concentrations are increased in alcohol-dependent men and women during early withdrawal, and these levels gradually decrease over time during abstinence [26, 27]. Orexin levels are positively correlated with alcohol craving, depression, and anxiety in humans [28], and administration of orexin directly into the brain increases anxiety-like behaviors in mice, rats, and hamsters [29–32]. Traditionally, OX1Rs have been considered to play a role in reward seeking, with OX2Rs playing more of a functional role in arousal [33, 34]. More recent work utilizing systemic or whole-brain administration of OX2R antagonists report reduced alcohol self-administration, but not cue-induced reinstatement, in mice and rats [35–37], suggesting that both receptors may be involved in voluntary alcohol consumption, although inhibition of VTA OX2Rs specifically does not significantly impact drinking in mice [38]. Because several studies implicate VTA OX1R signaling specifically in behavioral response to drugs of misuse (e.g., [39–42]), the focus of the current project was to examine *Hcrtr1* (OX1R) expression in the VTA and subregions of the extended amygdala during withdrawal from chronic alcohol exposure in adolescent and adult female rats.

## Methods

### Animals

All procedures were approved by the Institutional Animal Care and Use Committee of the Louisiana State University Health Sciences Center and were carried out in accordance with the National Institutes of Health guidelines. Female Wistar rats were obtained from Charles River Laboratories (Raleigh, NC, United States). Adolescent females were received at postnatal day 23 and triple-housed; adult females were received at postnatal day ∼56 and double-housed. In Experiment 3, we performed pilot studies in adult female Long Evans rats (Charles River Laboratories); because no differences between Wistar and Long Evans data were found (Figure 4; Supplementary Table S3), combined Long Evans and Wistar data are shown. All rats were maintained in a humidity- and temperature-controlled (22°C) vivarium on a 12-hour light/dark cycle (lights off at 7 AM), with *ad libitum* access to food and water. All animals were acclimated to housing conditions and light/dark cycles for 1 week before the start of experiments. A total of 78 rats (adolescent: n = 38; adults: n = 40) were used across experiments.

### Chronic intermittent exposure to alcohol vapor

In adolescent and adult female rats, a chronic intermittent exposure to alcohol vapor model was used to induce alcohol dependence. Rats were triple-housed (adolescent) or pair-housed (adult) in sealed chambers (La Jolla Alcohol Research, La Jolla, CA, United States) and exposed to alcohol vapor for 14 h/day. Blood alcohol levels (BALs) were measured 1–2 times weekly and analyzed using an Analox AM1 analyzer (Analox Instruments, Lunenberg, MA, United States). Adolescent females were exposed to vapor (AIE; adolescent intermittent exposure) for 4 weeks, and adult females were exposed to CIE for 6 weeks. The exposure time for adolescent rats was limited in order to restrict alcohol exposure to the adolescent time period. All rats were maintained within a BAL range of 150–250 mg/dL over the experimental time course (adolescents: BAL = 222 ± 9.1 mg/dL; adults: 164 ± 5.8 mg/dL; mean ± SEM). Behavioral testing and sacrifice time point all occurred during acute withdrawal (6–8 h after vapor cessation, while BALs were at or near 0 mg/dL).

### Surgeries

In Experiment 3, adult rats were anesthetized with isoflurane and mounted into a stereotaxic frame (Kopf Instruments, Tujunga, CA, United States). Custom 26 Ga bilateral cannulae (1.5 mm spacing, cut 9 mm below pedestal) were obtained from Plastics One (Roanoke, VA, United States). Cannulae were implanted above the VTA using the following coordinates from Bregma: −5.75 mm AP, +0.75 mm ML, −7.2 mm DV; injectors extended 1 mm below the pedestal for a final DV depth of −8.2 mm. Cannulae were secured to the skull with cranioplastic cement and stainless-steel anchor screws. After all surgical procedures, rats were monitored to ensure full recovery from anesthesia before being singly housed for one night. Rats were treated with the analgesic Meloxicam SR (4 mg/kg, s.c.) and the antibiotic cefazolin (20 mg/kg, i.m.) just prior to surgery. Weight and appearance were also monitored for a period of 7 d following surgery to detect any signs of distress or postoperative complications. Rats used for behavioral experiments were allowed 1 week to recover from any surgical procedure before the resumption of behavioral testing.

### Behavior

#### Hargreaves test for thermal nociception

Hargreaves testing was performed in adolescent females during the withdrawal period (6–8 h after vapor turned off). Briefly, rats were allowed to acclimate for 30 min prior to each testing session, then placed in a Plexiglas enclosure (4 × 8 × 5″) on top of a glass panel raised 8″ above the tabletop. Each hindpaw was stimulated by a halogen light heat source from an IITC model 309 Hargreaves apparatus (75% of maximum light intensity; IITC Life Sciences, Woodland Hills, CA, United States). Latency to withdraw the hindpaw was measured twice for each hindpaw in alternating order, with a minimum of 1 min between measurements. A 20-s cutoff was used to prevent tissue damage in non-responsive subjects. Measurements across the 4 trials were averaged as the thermal nociception score for each rat. We have previously published Hargreaves behavioral data shown in adolescent females in Experiment 1 [43]; in the present study, we examined the brains of these experimental animals.

#### Elevated plus maze

Elevated plus maze (EPM) testing was performed in adult females during the withdrawal period (6–8 h after vapor turned off) in Experiment 2. Rats were allowed to acclimate for 30 min prior to testing, then placed in a black Plexiglas EPM apparatus, raised 50 cm from the floor and consisting of 10 × 25 cm arms, two of which were enclosed by walls 20 cm high. The lighting measured at the center of the maze was adjusted to ∼100 lux. Rats were placed in the center of the maze and allowed 5 min to explore the apparatus. Time spent in open arms was calculated as a percentage of the time spent in the open arms divided by the total time in the open and closed arms (omitting center time).

In a separate cohort of alcohol-naïve rats, anxiety-like behavior was evaluated following intra-VTA administration of orexin A (Tocris Bioscience, Minneapolis, MN, United States) or vehicle (Experiment 3). Orexin A (50 nM in aCSF) or vehicle (aCSF) was administered into the VTA through 33-gauge injectors over 2.5 min at a rate of 0.2 μL/min, and 1 additional minute was allowed for diffusion before injector removal. Animals underwent sham infusions on days preceding drug infusion to acclimate them to the infusion procedure. Behavioral testing occurred 5 min after VTA injection. Prior to sacrifice, rats received intra-VTA injections 200 nL Evans Blue, and brains were sectioned to verify proper injection site.

### RT qPCR analysis of *Hcrt*

Lateral hypothalamus (LH)-containing tissue punches were taken from a separate cohort of AIE-exposed female Wistar rats, and total RNA was isolated using the miRNeasy Micro Kit (Qiagen, Hilden, Germany; Cat# 217084). Traces of contaminating DNA were removed using the DNA-free™ Kit following the manufacturer’s protocol (Invitrogen, Waltham, MA; Cat# AM1906). RNA concentration was measured with the NanoDrop^®^ ND-1000 UV/Vis Spectrophotometer (ThermoFisher Scientific, Carlsbad, California). cDNA synthesis was performed using the High-Capacity cDNA Reverse Transcription Kit (ThermoFisher Scientific, Cat# 4368814). Briefly, 10 µL containing 40 ng of RNA were mixed with 10 µL of 2X Reverse Transcription Buffer without RNAse inhibitor and incubated in a thermocycler (PTC-100, Bio-Rad, Hercules, CA, United States) at 25°C for 10 min, 37°C for 120 min, and 85°C for 5 min. TaqMan™ Gene Expression Assay (FAM, ThermoFisher Scientific, Cat# 4331182) was used for quantitative real-time PCR analysis (qPCR) using 20 µL of a reaction containing 10 µL of TaqMan™ Universal Master Mix II, 1 µL of the specific primer targeting Hypocretin neuropeptide precursor (*Hcrt*, Rn00565995), 2 µL of the cDNA, and 7 µL of RNase-free water. Cycling conditions were 50°C for 2 min and 95°C for 10 min, followed by 44 cycles of 95°C for 15 s and 60°C for 1 min. A melt curve analysis was conducted by heating to 95°C, followed by gradual 0.5°C decrements down to 65°C to confirm the specificity of amplification. Each reaction was duplicated without template controls (NTC) or reverse transcriptase controls. Peptidylprolyl isomerase A (*Ppia*, Rn00690933) served as the housekeeping gene for normalization. PCR data was analyzed using the 2^−ΔΔCT^ method, and results are presented as relative mRNA expression levels.

### *In situ* hybridization

RNAscope was performed in fresh frozen tissue from AIE or CIE-exposed tissue (Experiments 1 and 2) to evaluate gene expression patterns in the VTA, CeA, BNST, and NAc_shell_. Rats were sacrificed during acute withdrawal from alcohol (6–8 h after vapor turned off), and brains were snap-frozen is isopentane and stored at −80°C until sectioning. A randomized strategy was used to select a subset of AIE/CIE and alcohol-naive brains for RNAscope analysis. Ten µm-thick coronal sections containing the NAc_shell_, BNST, CeA, and VTA were collected using a cryostat, mounted onto Superfrost glass slides, and stored at −80°C until the assay was performed. Each brain region was analyzed at the same approximate depth along the anterior-posterior axis for each rat, which are as follows: NAc_shell_, Bregma +1.20 mm; BNST, Bregma +0.00 mm; CeA, Bregma −2.40 mm; VTA, Bregma −5.88 mm.

*In situ* hybridization was performed using the RNAscope^®^ Fluorescent Multiplex Reagent kit v1 following manufacturer’s recommendations (ACDBio, Newark, CA, United States) and as previously described [43, 44]. Briefly, slides were fixed in chilled 4% PFA then dehydrated using progressive ethanol washes. Sections were incubated for 30 min with Protease III at room temperature, washed in PBS, then incubated with probes for 2 h at 40°C. NAc_shell_-, BNST-, and CeA-containing tissue sections were incubated with probes targeting *Hcrtr1* (Rn-Hcrtr1, Cat#. 444761), *Drd1* (Rn-Drd1a-C2, Cat# 317031-C2), and *Fos* (Rn-Fos-C3, Cat# 403591-C3). VTA-containing sections were incubated with probes targeting *Hcrtr1* (Rn-Hcrtr1, Cat#. 444761), *Th* (Rn-Th-C2, Cat# 314651-C2), and *Fos* (Rn-Fos-C3, Cat# 403591-C3). Sections were then washed and incubated with amplification reagents, per manufacturer instructions. Each time RNAscope was performed, a separate tissue section was incubated with a negative control probe (Cat# 320871), which was analyzed in order to determine the threshold for counting a positive signal in each channel [44]. Sections were coverslipped with Fluoro-Gel II with DAPI (Electron Microscopy Sciences, Hatfield, PA, United States; Cat# 17985-51) and imaged using a Zeiss AxioScan Z.1 slide scanner (Oberkochen, Germany) with a ×20 objective yielding images with a 0.22 μm/pixel resolution.

Images were analyzed using Qupath 0.4.1 [45] by quantifying the total number of puncta in each cell for each channel within a selected region of interest. Negative control probe-processed tissue was used to establish a threshold (defined as the sum of the mean number of puncta per cell and the standard deviation) for each channel. Only cells exceeding this threshold in processed tissue were counted as expressing mRNA of interest.

### Statistical analysis

Statistical analysis was performed in Prism version 9.4.1 (GraphPad Software, La Jolla, CA, United States). Data were analyzed using two-tailed *t*-tests and linear regression, except where otherwise indicated. In Figure 2, a Mann-Whitney U-test was performed due to non-normal distribution of data. A *p* value of <0.05 was considered significant.

## Results

### Adolescent intermittent exposure (AIE) to ethanol

**FIGURE 1:**
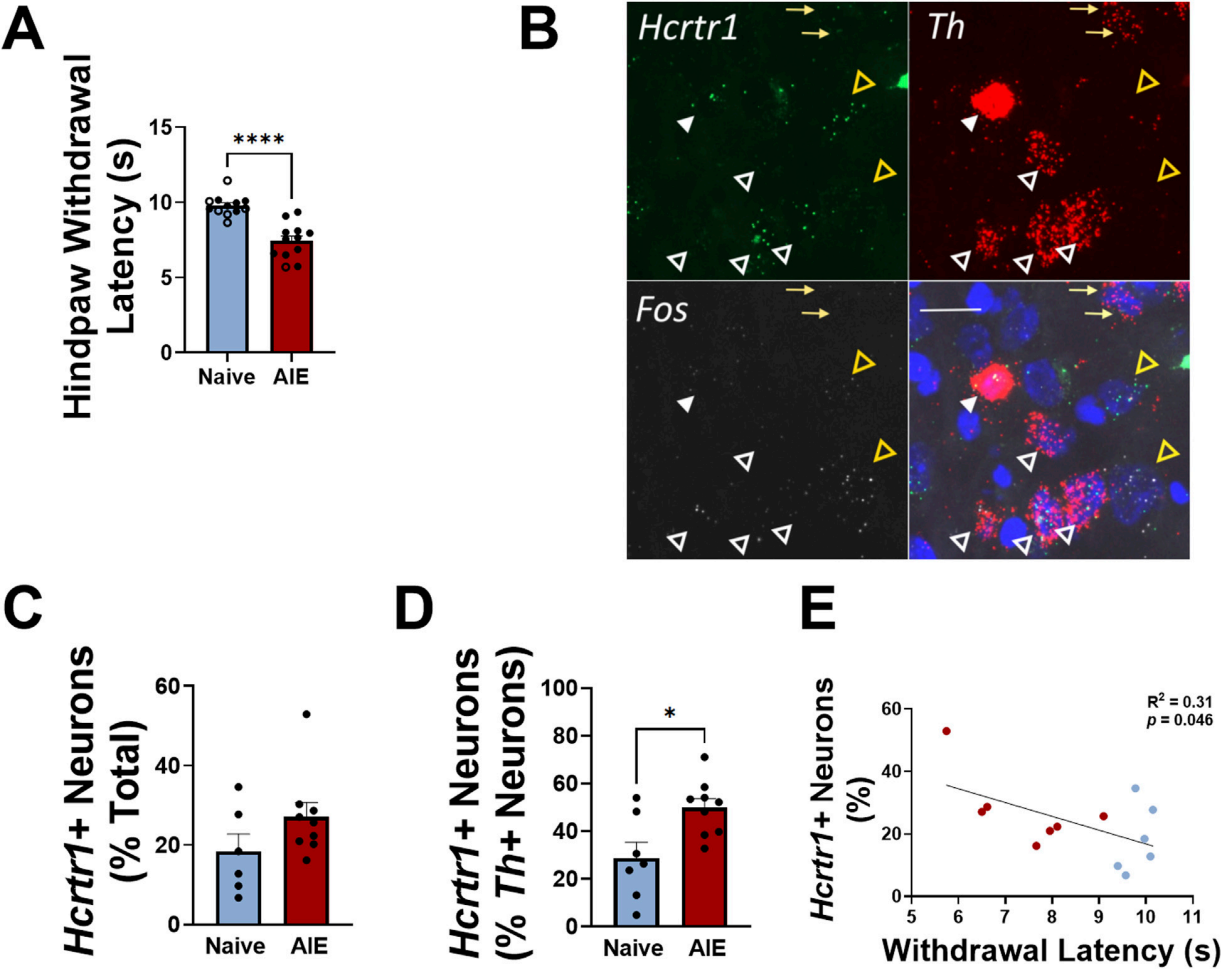
Adolescent females exhibit thermal hypersensitivity during alcohol withdrawal that is positively correlated with VTA *Hcrtr1* expression. **(A)** Hindpaw withdrawal latency during Hargreaves testing in alcohol-naïve (blue) or AIE (red) female rats tested during acute withdrawal. Closed circles indicate rats used for tissue analysis; open circles indicate rats used for behavioral testing, but not tissue analysis. Reproduced with permission from “Figure 1. Intermittent alcohol exposure during adolescence produces sex-dependent long-lasting changes in pain sensitivity. (A) Timeline of the experiment including adolescence intermittent alcohol exposure (AIE), chronic withdrawal and pain-like behaviors from adolescent to early adulthood. Hargreaves (B) and Von Frey (C) thresholds in male rats during acute (∼7 hours after each daily exposure) and chronic alcohol withdrawal intervals. Control animals (AIR) are shown in open circles while exposed animals (AIE) are shown in closed purple circles. Hargreaves (D) and Von Frey (E) thresholds in female rats during acute (∼7 hours after each daily exposure) and chronic alcohol withdrawal intervals. Control animals (AIR) are shown in open circles while exposed animals (AIE) are shown in closed green circles. The data are represented as the mean ± SEM (males: n = 96; females: n = 36): *p < 0.05 AIR vs. AIE was assessed by two-way analysis of variance with repeated measures followed by Sidak’s post hoc test.” by Maria E. Secci, Leslie K. Kelley, Elizabeth M. Avegno, Eleanor B. Holmgren, Lily Chen, Sydney L. Rein, Sheila A. Engi, Virginia Quinlan, LisaWilson, Nicholas W. Gilpin, Tiffany A. Wills, licensed under CC BY-NC-ND. **(B)** Representative images of VTA *Hcrtr1* (green), *Fos* (white), and *Th* (red) expression. Bottom right panel shows merged image with DAPI (blue). Yellow arrows indicate *Th*+, *Hcrtr1*-, *Fos*- neurons; white closed arrowheads indicate *Th*+, *Hcrtr1*+, *Fos*- neurons; white open arrowheads indicate *Th*+, *Hcrtr1*+, *Fos*+ neurons; open yellow arrowheads indicate *Th*-, *Hcrtr1*+, *Fos*+ neurons. Scale bar, 20 µm. **(C)** Number of VTA *Hcrtr1*+ neurons, expressed as a percentage of all neurons (DAPI+) in alcohol-naïve and AIE rats. **(D)** Number of VTA *Hcrtr1*+ neurons, expressed as a percentage of all *Th*+ neurons, in alcohol-naïve and AIE rats. **(E)** Number of VTA *Hcrtr1* neurons plotted against hindpaw withdrawal latency for individual rats. Data in **(A, C, D)** shown as mean ± SEM; *p < 0.05; ****p < 0.0001; two-tailed *t*-test. Data in **(E)** analyzed using linear regression; R^2^ = 0.31; *p* = 0.046.

In Experiment 1, adolescent female rats were exposed to AIE from postnatal day 30 (p30)-p58, as previously reported [43]. Hargreaves testing for thermal nociception occurred during acute withdrawal from alcohol (6–8 h after vapor turned off); AIE-exposed females demonstrated a significantly lower latency to withdraw their hindpaw compared to air-exposed controls after 4 weeks of vapor exposure (*t*_22_ = 6.0, *p* < 0.0001, two-tailed *t*-test; Figure 1A). VTA-containing sections were used to evaluate expression of *Hcrtr1*, *Fos*, and *Th* (tyrosine hydroxylase, the enzyme involved in the rate limiting step of dopamine synthesis, and marker of dopamine neurons in the VTA; Figure 1B), and NAc_shell_-, BNST-, and CeA-containing sections were used to evaluate expression of *Hcrtr1*, *Fos*, and *Drd1* (dopamine receptor 1; data not shown). When comparing tissue from AIE females to naïve controls in each brain region, we did not observe a significant increase in total *Hcrtr1*+ cells in the VTA of AIE rats compared to alcohol-naïve controls (expressed as a percentage of all DAPI+ neurons; Figure 1C), although we found a significantly greater number of *Hcrtr1*+ cells (expressed as a percentage of all *Th*+ neurons in the VTA; *t*_14_ = 2.9, *p* = 0.012, two-tailed *t*-test; Figure 1D). Approximately 25% of *Hcrtr1*+ VTA neurons expressed *Th*+, with no significant difference between AIE and alcohol-naïve control rats (25.1% ± 5.8% in alcohol-naïve controls; 24.5% ± 1.8% AIE; *t*_14_ = 0.11, *p* = 0.92, two-tailed *t*-test). No significant difference in *Fos* expression was observed in the VTA (*t*_14_ = 0.38, *p* = 0.71, two-tailed *t*-test), and no significant difference in expression of any markers analyzed in the extended amygdala brain regions was found (Supplementary Table S1), indicating region-specific changes in *Hcrtr1* expression in AIE rats.

**FIGURE 2:**
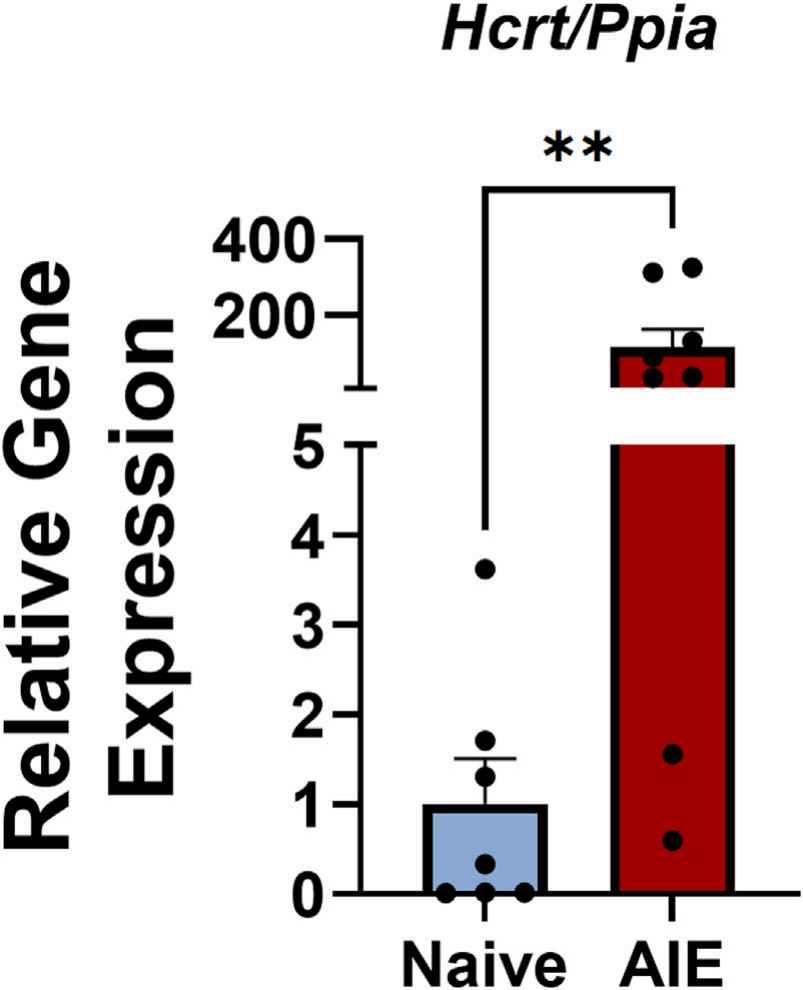
Adolescent females exhibit higher *Hcrt* expression in the LH during alcohol withdrawal relative to alcohol-naïve controls. Expression levels of *Hcrt* (relative to the housekeeping gene *Ppia*) in LH samples from alcohol-naïve (blue) and AIE (red) rats. Data shown as mean ± SEM; **p = 0.0059; Mann-Whitney U-test.

We next plotted the number of VTA *Hcrtr1*+ cells against hindpaw withdrawal latency and found a significant negative correlation between the two (R^2^ = 0.31, *p* = 0.046, linear regression analysis; Figure 1E). In tissue samples taken from a separate cohort of AIE or alcohol-naïve females, we performed qPCR analysis of *Hcrt* (hypocretin; orexin) expression in the lateral hypothalamus (LH) and found significantly higher expression of *Hcrt* in the LH of AIE females compared to alcohol-naïve controls (*U* = 5, *p* = 0.0059, Mann-Whitney U-test; Figure 2). Collectively, these data indicate region-specific alterations within the orexin signaling system following adolescent alcohol exposure, as well as a relationship between VTA *Hcrtr1* expression and thermal hyperalgesia that manifests during alcohol withdrawal.

### Chronic intermittent exposure (CIE) to ethanol during adulthood

**FIGURE 3:**
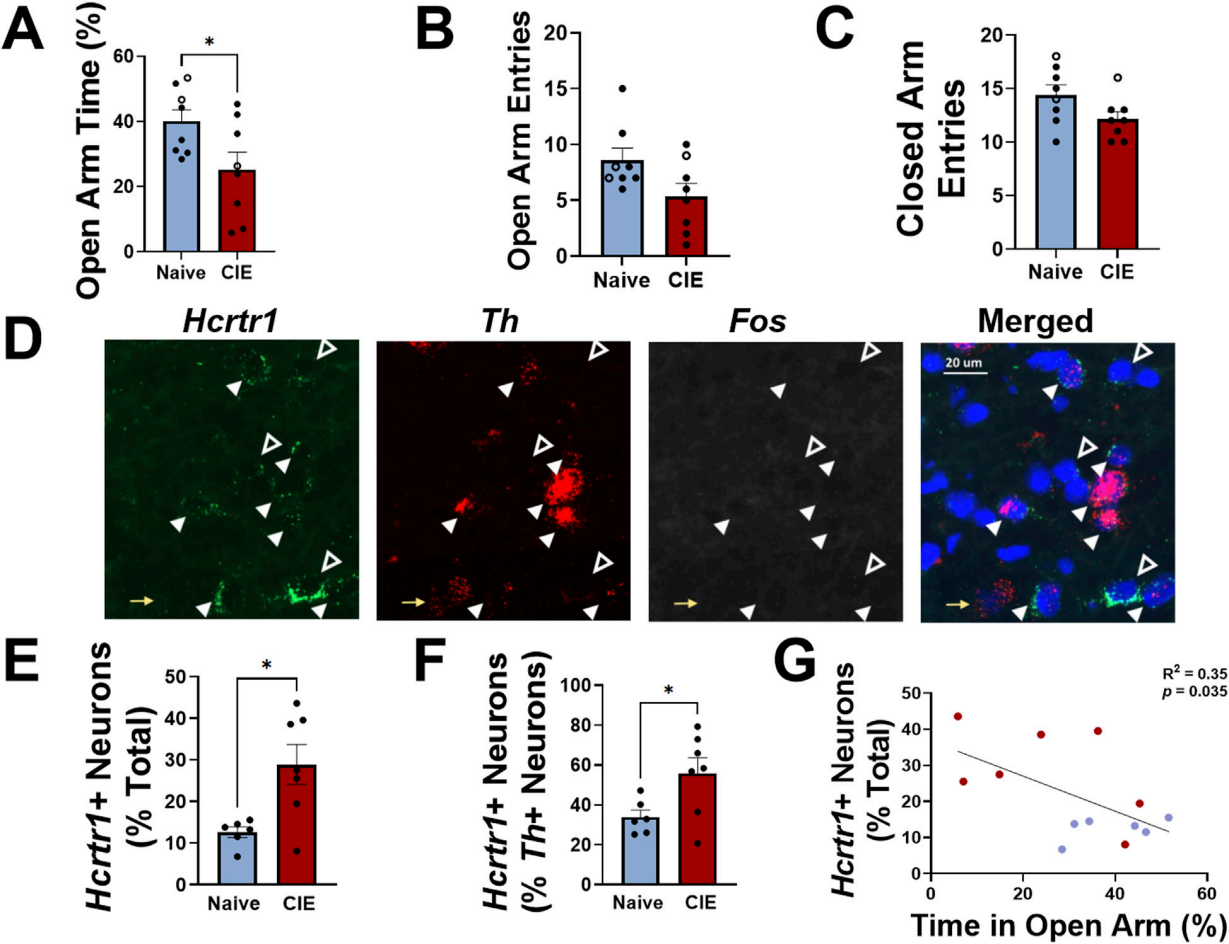
Adult females exhibit higher anxiety-like behavior than alcohol-naïve controls during alcohol withdrawal that is positively correlated with VTA *Hcrtr1* expression. **(A)** Time spent in the open arm of an elevated plus maze (expressed as a percentage of total open and closed arm time) in alcohol-naïve (air-exposed, blue) or CIE (red) female rats tested during acute withdrawal. Total number of open **(B)** and closed **(C)** arm entries shown for both groups. Closed circles in **(A–C)** indicate rats used for tissue analysis; open circles indicate rats used for behavioral testing, but not tissue analysis. **(D)** Representative images of VTA *Hcrtr1* (green), *Fos* (white), and *Th* (red) expression. Right panel shows merged image with DAPI (blue). Yellow arrows indicate *Th*+, *Hcrtr1*-, *Fos*- neurons; white closed arrowheads indicate *Th*+, *Hcrtr1*+, *Fos*- neurons; white open arrowheads indicate *Th*-, *Hcrtr1*+, *Fos*- neurons. Scale bar, 20 µm. **(E)** Number of VTA *Hcrtr1*+ neurons, expressed as a percentage of all neurons (DAPI+) in alcohol-naïve and CIE rats. **(F)** Number of VTA *Hcrtr1*+ neurons, expressed as a percentage of all *Th*+ neurons, in alcohol-naïve and CIE rats. **(G)** Number of VTA *Hcrtr1* neurons plotted against EPM open arm time for individual rats. Data in **(A–C)**; **E, F** shown as mean ± SEM; *p < 0.05; two-tailed *t*-test. Data in **(G)** analyzed using linear regression; R^2^ = 0.35; *p* = 0.035.

In Experiment 2, female rats were exposed to CIE for 6 weeks throughout adulthood (beginning ∼9 weeks of age). Anxiety-like behavior was assessed using the elevated plus maze (EPM) during acute withdrawal from alcohol (6–8 h after vapor turned off); CIE-exposed females demonstrated a significantly lower time in the open arm of the EPM compared to alcohol-naïve controls (expressed as a percentage of total open+closed arm time; *t*_14_ = 2.29, *p* = 0.038, two-tailed *t*-test; Figure 3A). Total number of open arm entries was not significantly reduced in CIE rats compared to alcohol-naïve controls (*t*_14_ = 2.1, *p* = 0.056, two-tailed *t*-test; Figure 3B), and no difference between closed arm entries was observed between groups (*t*_14_ = 1.9, *p* = 0.075, two-tailed *t*-test; Figure 3C). VTA-containing sections were used to evaluate expression of *Hcrtr1*, *Fos*, and *Th* (Figure 3D), and NAc_shell_-, BNST-, and CeA-containing sections were used to evaluate expression of *Hcrtr1*, *Fos*, and *Drd1*. When comparing tissue from CIE females to naïve controls in each brain region, we observed a significantly greater number of *Hcrtr1*+ cells in the VTA of CIE rats compared to alcohol-naïve controls (expressed as a percentage of all DAPI+ neurons; *t*_11_ = 3.07, *p* = 0.011; Figure 3E), as well as a significantly greater number of *Hcrtr1*+ cells (expressed as a percentage of all *Th*+ neurons in the VTA; *t*_11_ = 2.4, *p* = 0.034, two-tailed *t*-test; Figure 3F). No significant difference in the number of *Hcrtr1*+ VTA neurons that expressed *Th* was observed between CIE and alcohol-naïve control rats (29.5% ± 6.3% in alcohol-naive controls; 40.7 ± 8.0% CIE; *t*_11_ = 1.1, *p* = 0.29, two-tailed *t*-test), and no significant difference in expression of any other markers was found in the VTA or other brain regions analyzed (Supplementary Table S2), indicating region-specific changes in *Hcrtr1* expression in CIE rats. We next plotted the number of VTA *Hcrtr1*+ cells against open arm time in the EPM and found a significant negative correlation between the two (R^2^ = 0.35, *p* = 0.035, linear regression analysis; Figure 3G). These data indicate a difference in *Hcrtr1* expression in CIE rats compared to alcohol-naïve controls that is limited to the VTA, as well as a relationship between VTA *Hcrtr1* expression and increased anxiety-like behavior that manifests during alcohol withdrawal.

### Intra-VTA administration of orexin A

**FIGURE 4:**
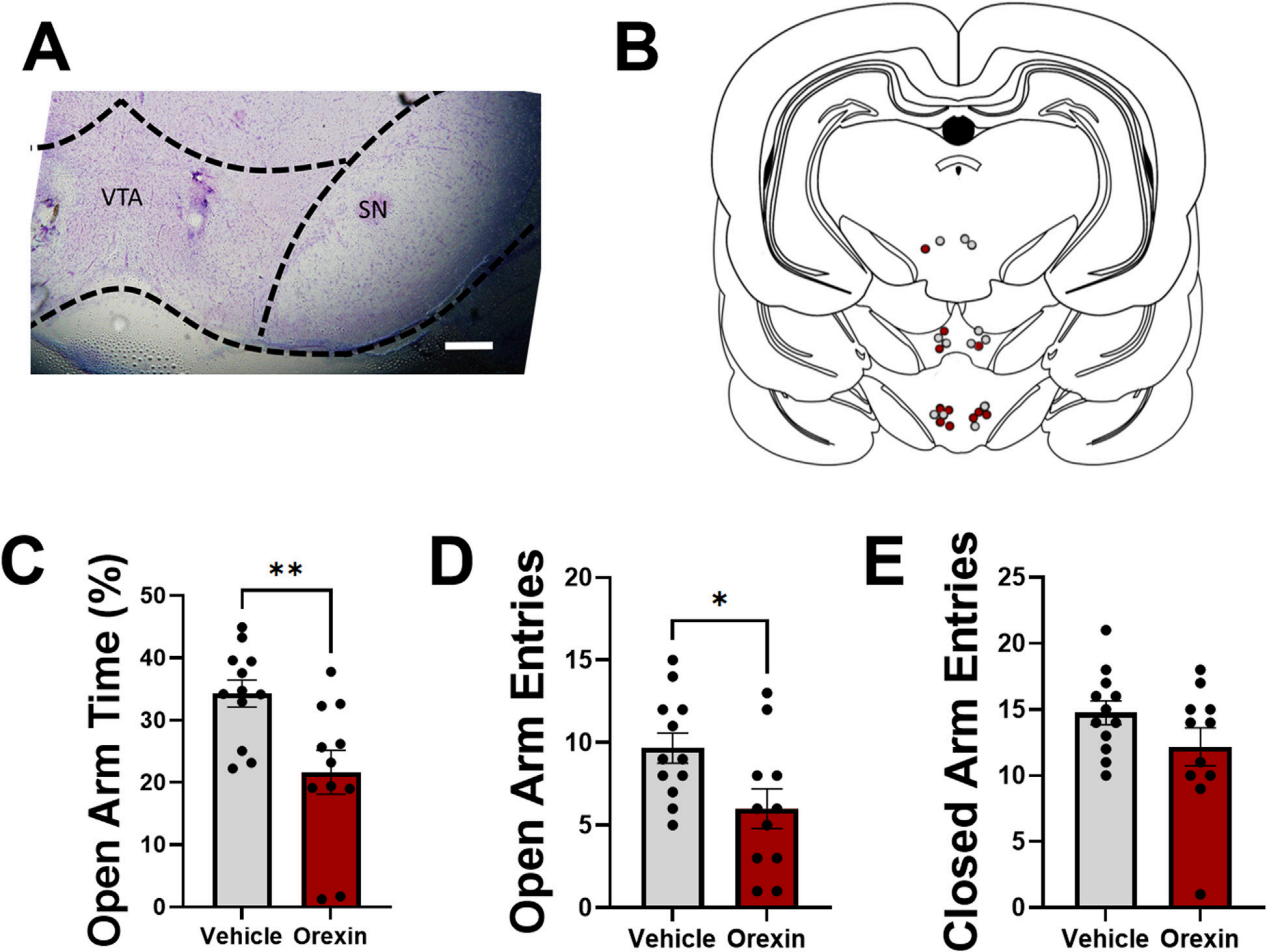
Intra-VTA administration of orexin-A increases anxiety-like behavior in alcohol-naïve adult female rats. **(A)** Representative injection site demonstrating cannula placement in VTA. Substantia nigra (SN) indicated for reference. Scale bar, 500 µm. **(B)** Map indicating representative injection sites of aCSF (grey) or orexin A (red) for all experimental rats. Bilateral injections were performed, although only one injection site is shown per rat for simplification. **(C)** Time spent in the open arm of an elevated plus maze (expressed as a percentage of total open and closed arm time) in rats following intra-VTA administration of vehicle (aCSF; grey) or orexin A (50 nM; red). Total number of open **(D)** and closed **(E)** arm entries shown for both groups. Data in **(C–E)** shown as mean ± SEM; *p < 0.05, **p < 0.01, two-tailed *t*-test.

In Experiment 3, we sought to determine whether intra-VTA administration of orexin A in otherwise experimentally naïve females could produce an anxiety-like phenotype observed in CIE females tested during withdrawal. In a separate cohort of adult female rats, guide cannulae were positioned above the VTA, and 500 nL injections of orexin A (50 nM in aCSF) or vehicle (aCSF) were delivered 5 min prior to behavioral testing in an EPM (Figures 4A, B). Intra-VTA orexin A administration significantly reduced time spent in the open arm of an EPM (*t*_21_ = 3.1, *p* = 0.0052, two-tailed *t*-test; Figure 4C), as well as the total number of open arm entries (*t*_21_ = 2.5, *p* = 0.023, two-tailed *t*-test; Figure 4D), suggesting that activation of VTA orexin receptors is sufficient to produce an anxiety-like phenotype similar to what is observed during withdrawal from chronic alcohol. Total number of closed arm entries did not differ between groups (*t*_21_ = 1.5, *p* = 0.14, two-tailed *t*-test; Figure 4E), indicating that intra-VTA orexin A administration did not reduce general locomotor activity in the EPM.

## Discussion

These results indicate a higher number of *Hcrtr1*-expressing neurons specifically in the VTA of female rats following chronic alcohol exposure, which is correlated with two separate alcohol withdrawal-induced behaviors, during adolescence and adulthood. These changes were not observed in regions within the extended amygdala, including the NAc_shell_, BNST, and CeA, indicating region-specific alterations in *Hcrtr1* expression.

In humans with AUD, higher levels of circulating orexin are found during early withdrawal, which decrease over 4 weeks of abstinence [26, 27], and a positive correlation between orexin levels and negative affective symptoms including depression and anxiety has been reported in humans [28]. Administration of orexin in brain produces a high-anxiety phenotype in mice, rats, and hamsters [29–32], providing additional preclinical support for a role of orexin signaling in anxiogenic behavior. Here, we demonstrate an increase in VTA *Hcrtr1* expression following chronic alcohol exposure that is significantly correlated with anxiety-like behavior, suggesting that higher levels of OX1R expression (and potentially greater VTA OX1R activation) may be linked to higher anxiety-like behavior. Additionally, we demonstrate that intra-VTA orexin A administration alone can produce an anxiety-like phenotype in otherwise experimentally naïve adult female rats. Because orexin A can bind to both OX1R and OX2Rs, and both OX1R and OX2R are expressed in the VTA [25], we cannot rule out the possibility that intra-VTA orexin A administration produces an anxiogenic effect via actions on OX2Rs. Acute i.c.v. administration of orexin A administration elicits a stress response in rats [46, 47] and an anxiogenic phenotype in mice and rats [31], although the specific brain regions involved in these behaviors are not defined. Our data demonstrate a role of the VTA in anxiogenic effects of orexin A specifically. Whether increased activation of VTA OX1Rs causes an anxiogenic phenotype during withdrawal from chronic alcohol remains to be determined, but our data do point to a relationship between the VTA orexin system and anxiety-like behavior.

The relationship between orexin and nociception is less straightforward, however. Preclinical studies have shown an analgesic effect of intravenous administration of orexin A in mice and rats via actions at the level of the spinal cord [48], and in a model of neuropathic pain, LH stimulation decreased thermal nociception in female rats via OX1R signaling [49], suggesting that orexin A can produce an antinociceptive effect by binding to OX1Rs in certain areas. Systemic administration of a dual OX1/OX2R antagonist reduced increased thermal and mechanical nociception in adult male C57BL/6J mice in a model of chronic predictable mild stress [50], indicating that blockade of OX1R signaling can also have an antinociceptive effect. These conflicting results may be due to the location of OX1Rs and potentially the preclinical model used to induce a pain-like phenotype. Here, we demonstrate an increase in *Hcrt* in the LH of female AIE rats compared to alcohol-naïve naïve controls, an increase in VTA *Hcrtr1+, Th*+ neurons, and a negative correlation between VTA *Hcrtr1* expression and hindpaw withdrawal latency. While these data do not allow us to draw causal conclusions, they do raise a potential relationship between orexin signaling at the level of the VTA and heightened nociception during alcohol withdrawal. Future research can test this relationship directly by determining whether inhibition of VTA OX1Rs can rescue heightened thermal nociception in AIE rats compared to alcohol-naïve controls and/or by evaluating whether intra-VTA orexin administration can produce a hyperalgesia phenotype in otherwise experimentally naïve rats.

While our *in situ* hybridization analysis included multiple gene targets across several brain regions, the significant differences found were limited to VTA *Hcrtr1* expression only, indicating regional specificity in expression of this gene. We did not observe changes in VTA *Th* expression or *Drd1* expression in NAc_shell_, BNST, or CeA, and we did not observe changes in *Fos* expression in any region. We have previously demonstrated increased Fos protein expression in the VTA of CIE male rats during withdrawal [22], although it is likely that acute withdrawal (6–8 h after last exposure) is too diffuse a time point to observe changes in mRNA expression. Indeed, *Fos* mRNA expression typically peaks shortly after exposure to a stimulus and decreases within 1–2 h, while Fos protein expression persists for several hours after stimulus exposure [51]. Whether the changes in *Hcrtr1* expression translate to changes in protein expression or function remains to be determined.

While VTA OX1R signaling has been implicated in behavioral response to other drugs of misuse, including cocaine, morphine, and methamphetamine [39–41], less is known about VTA OX1R and its relationship to behavioral dysregulation following chronic alcohol specifically. Collectively, our data provide evidence of altered *Hcrtr1* expression that is region specific and correlated with two separate alcohol withdrawal-associated behaviors (thermal hyperalgesia and anxiety-like behavior) following chronic alcohol exposure at two separate developmental timepoints (adolescence and adulthood, respectively). Whether these relationships are maintained throughout development (i.e., whether VTA *Hcrtr1* expression correlates with anxiety-like behavior during adolescence and thermal nociception in adulthood) remains to be determined. These data raise the possibility that VTA orexin signaling plays a role in mediating alcohol withdrawal-associated behaviors. Ongoing and future experiments will bridge these observations by determining whether orexin modulation of VTA neurons and/or LH-VTA circuit activity is necessary for these behaviors during WD from chronic alcohol, and whether these observations are found in male rats as well.

## Data Availability

The raw data supporting the conclusions of this article will be made available by the authors, without undue reservation.
