# Supplementary material for: Chronic alcohol withdrawal-associated increases in VTA Hcrtr1 expression are associated with heightened nociception and anxiety-like behavior in female rats
Source: Adv Drug Alcohol Res. 2025 Apr 3;5:14199. doi: 10.3389/adar.2025.14199 (PMC12004375; doi:10.3389/adar.2025.14199)
Supplement: Supplementary file 1 [file DataSheet1.docx]

| **Brain Region** | ***Hcrtr1*+ Neurons (% of total)** | | ***Drd1*+ Neurons (% of total)** | | ***Hcrtr1*+ Neurons (% of *Drd1*+)** | | ***Drd1*+ Neurons (% of *Hcrtr1*+)** | | ***Fos*+ Neurons (% of *Drd1*+)** | | ***Fos*+ Neurons (% of *Hcrtr1*+)** | |
| --- | --- | --- | --- | --- | --- | --- | --- | --- | --- | --- | --- | --- |
|  | **Control** | **AIE** | **Control** | **AIE** | **Control** | **AIE** | **Control** | **AIE** | **Control** | **AIE** | **Control** | **AIE** |
| **NAc medial shell** | 14.2 (3.1) | 7.4 (1.5) | 38.3 (4.6) | 36.0 (2.3) | 17.5 (4.5) | 9.8 (2.3) | 44.4 (4.0) | 44.4 (2.8) | 18.9 (5.6) | 21.7 (4.7) | 25.5 (6.6) | 27.3 (6.3) |
| **NAc lateral shell** | 23.2 (5.6) | 14.4 (3.1) | 29.0 (4.1) | 33.1 (3.7) | 35.3 (9.9) | 21.7 (5.0) | 39.1 (3.7) | 49.5 (8.1) | 21.2 (3.8) | 28.4 (10.1) | 28.3 (6.7) | 34.6 (11.9) |
| **Total NAc shell** | 14.5 (2.7) | 9.8 (1.7) | 36.9 (3.2) | 35.9 (1.8) | 17.0 (3.6) | 12.9 (2.5) | 41.4 (2.4) | 44.6 (2.5) | 17.4 (4.5) | 22.2 (4.7) | 22.2 (5.7) | 25.9 (4.6) |
| **Dorsal BNST** | 51.0 (5.1) | 46.5 (4.7) | 24.4 (2.4) | 25.0 (2.7) | 56.0 (5.4) | 53.6 (6.2) | 26.8 (2.4) | 28.1 (2.9) | 24.2 (5.7) | 21.4 (3.0) | 21.8 (4.5) | 20.7 (3.5) |
| **Ventral BNST** | 52.9 (3.3) | 49.8 (3.6) | 23.3 (1.7) | 25.8 (2.8) | 66.9 (2.4) | 59.1 (5.1) | 29.6 (2.3) | 30.1 (3.3) | 27.7 (5.1) | 21.5 (2.2) | 23.1 (3.4) | 22.0 (3.0) |
| **Total BNST** | 51.7 (4.3) | 47.8 (4.4) | 23.9 (2.0) | 25.2 (2.6) | 59.2 (4.5) | 55.3 (5.8) | 27.4 (2.0) | 28.7 (2.8) | 25.2 (5.1) | 21.9 (2.4) | 22.3 (3.9) | 21.3 (3.1) |
| **Medial CeA** | 40.9 (5.1) | 36.0 (4.1) | 11.7 (1.9) | 9.1 (1.4) | 65.7 (9.6) | 56.6 (7.7) | 18.9 (3.6) | 13.2 (1.7) | 42.4 (9.6) | 52.5 (5.5) | 40.8 (8.9) | 49.9 (5.9) |
| **Lateral CeA** | 42.8 (4.0) | 35.3 (4.8) | 10.2 (3.5) | 8.2 (1.7) | 57.5 (7.4) | 49.3 (7.0) | 14.1 (4.7) | 11.3 (2.4) | 53.6 (4.2) | 58.0 (6.9) | 43.8 (6.4) | 54.9 (4.0) |
| **Total CeA** | 42.1 (4.3) | 35.4 (3.3) | 10.8 (1.8) | 8.5 (1.1) | 65.6 (9.1) | 59.0 (3.1) | 16.1 (2.0) | 13.8 (1.5) | 46.1 (7.4) | 55.0 (4.9) | 42.4 (7.1) | 52.8 (4.8) |

**Supplementary Table 1**: Neuronal expression patterns in extended amygdala subregions of adolescent female rats exposed to chronic alcohol (AIE) or ambient air (control). Table quantifies total number of *Hcrtr1*+ and *Drd1*+ cells (expressed as a percentage of all neurons); number of *Hcrtr1*+ neurons (as a percentage of all *Drd1*+ cells); number of *Drd1*+ neurons (as a percentage of all *Hcrtr1*+ cells); and number of *Fos*+ neurons (as a percentage of all *Drd1*+ cells or as a percentage of all *Hcrtr1*+ cells). Data is shown for NAc_shell_, BNST, and CeA, as well as subdivisions (medial/lateral or dorsal/ventral) of each brain region. Mean values shown, with SEM in parentheses. Two-tailed *t*-tests revealed no significant differences between AIE and controls for each gene in each region.

| **Brain Region** | ***Hcrtr1*+ Neurons (% of total)** | | ***Drd1*+ Neurons (% of total)** | | ***Hcrtr1*+ Neurons (% of *Drd1*+)** | | ***Drd1*+ Neurons (% of *Hcrtr1*+)** | | ***Fos*+ Neurons (% of *Drd1*+)** | | ***Fos*+ Neurons (% of *Hcrtr1*+)** | |
| --- | --- | --- | --- | --- | --- | --- | --- | --- | --- | --- | --- | --- |
|  | **Control** | **CIE** | **Control** | **CIE** | **Control** | **CIE** | **Control** | **CIE** | **Control** | **CIE** | **Control** | **CIE** |
| **NAc medial shell** | 12.7 (5.5) | 11.9 (1.3) | 25.5 (4.9) | 21.1 (1.4) | 22.8 (8.3) | 22.6 (3.3) | 49.9 (11.3) | 40.0 (2.0) | 38.1 (10.8) | 64.2 (6.0) | 46.0 (12.4) | 72.5 (4.7) |
| **NAc lateral shell** | 20.5 (2.6) | 20.8 (5.2) | 30.4 (4.0) | 27.7 (2.9) | 39.0 (7.1) | 34.8 (8.8) | 39.1 (3.7) | 49.5 (8.1) | 39.0 (11.5) | 68.8 (6.1) | 42.8 (11.1) | 70.8 (7.8) |
| **Total NAc shell** | 16.1 (4.3) | 15.2 (2.6) | 28.3 (4.6) | 21.1 (2.6) | 28.9 (6.4) | 28.9 (5.1) | 53.0 (10.1) | 37.2 (3.6) | 41.1 (10.7) | 58.1 (8.3) | 46.9 (11.8) | 62.2 (7.1) |
| **Dorsal BNST** | 24.3 (4.3) | 19.9 (2.6) | 16.3 (3.0) | 17.2 (3.1) | 51.5 (7.3) | 64.2 (8.0) | 32.1 (9.5) | 34.8 (4.9) | 21.7 (8.3) | 26.5 (6.5) | 13.4 (5.5) | 10.2 (3.9) |
| **Ventral BNST** | 20.2 (2.7) | 21.4 (2.2) | 28.8 (3.9) | 28.1 (3.9) | 50.7 (4.9) | 59.1 (6.5) | 71.8 (6.0) | 73.9 (8.9) | 24.7 (8.4) | 28.0 (5.8) | 24.0 (7.3) | 31.7 (7.8) |
| **Total BNST** | 22.9 (3.7) | 20.4 (1.9) | 20.5 (3.1) | 21.3 (2.9) | 51.3 (5.7) | 60.0 (6.3) | 48.1 (7.6) | 60.0 (6.9) | 23.1 (8.3) | 26.5 (5.6) | 17.5 (6.1) | 24.2 (6.5) |
| **Medial CeA** | 45.5 (3.9) | 38.2 (5.0) | 23.0 (3.0) | 30.3 (3.5) | 94.4 (2.4) | 78.8 (8.5) | 47.7 (5.5) | 64.5 (7.0) | 48.6 (9.6) | 41.0 (10.7) | 38.1 (9.7) | 32.8 (8.7) |
| **Lateral CeA** | 35.7 (4.5) | 28.9 (4.5) | 20.2 (4.1) | 22.9 (2.4) | 46.8 (8.3) | 57.1 (6.6) | 80.8 (4.4) | 68.0 (7.4) | 51.6 (9.4) | 33.1 (9.3) | 46.3 (9.1) | 27.4 (9.0) |
| **Total CeA** | 40.5 (4.1) | 34.1 (3.9) | 21.6 (3.4) | 27.0 (2.8) | 87.8 (3.1) | 75.5 (7.2) | 47.0 (6.4) | 61.7 (6.8) | 50.0 (9.5) | 37.3 (9.7) | 41.5 (9.4) | 30.2 (8.8) |

**Supplementary Table 2**: Neuronal expression patterns in extended amygdala subregions of adult female rats exposed to chronic alcohol (CIE) or ambient air (control). Table quantifies total number of *Hcrtr1*+ and *Drd1*+ cells (expressed as a percentage of all neurons); number of *Hcrtr1*+ neurons (as a percentage of all *Drd1*+ cells); number of *Drd1*+ neurons (as a percentage of all *Hcrtr1*+ cells); and number of *Fos*+ neurons (as a percentage of all *Drd1*+ cells or as a percentage of all *Hcrtr1*+ cells). Data is shown for NAc_shell_, BNST, and CeA, as well as subdivisions (medial/lateral or dorsal/ventral) of each brain region. Mean values shown, with SEM in parentheses. Two-tailed *t*-tests revealed no significant differences between AIE and controls for each gene in each region.

| **Group** | **Vehicle-Treated** | | | **Orexin-Treated** | | |
| --- | --- | --- | --- | --- | --- | --- |
|  | **Wistar** | **Long Evans** | ***t*-test** | **Wistar** | **Long Evans** | ***t*-test** |
| **Open Arm Time** | 33.1 (3.1) | 36.5 (1.7) | *t*_10_ = 0.72; *p* = 0.49 | 24.3 (4.0) | 14.5 (6.7) | *t*_9_ = 1.29; *p* = 0.23 |
| **Open Arm Entries** | 9.5 (1.2) | 10.0 (1.6) | *t*_10_ = 0.25; *p* = 0.81 | 7.4 (1.4) | 2.3 (0.67) | *t*_9_ = 2.16; *p* = 0.059 |
| **Closed Arm Entries** | 15.4 (1.1) | 13.5 (1.7) | *t*_10_ = 0.98; *p* = 0.35 | 13.0 (1.9) | 10.0 (0.58) | *t*_9_ = 0.93; *p* = 0.38 |

**Supplementary Table 3**: Comparison of elevated plus maze data from Wistar and Long Evans females. Table quantifies open arm time, open arm entries, and closed arm entries for vehicle-treated and orexin-treated Wistar and Long Evans rats; data expressed as average values, with SEM in parenthesis. Two-tailed *t*-tests revealed no significant differences between vehicle- or orexin-treated Wistar and Long Evans rats for each metric
